# Strategy to small intestine obstruction caused by Crohn’s disease on the basis of transnasal ileus tube insertion

**DOI:** 10.1186/s12893-022-01632-w

**Published:** 2022-05-14

**Authors:** Lingyun Zuo, Lei Cao, Chengliang Ding, Hongfei Tu, Cheng Wei, Lili Yuan, Huali Wang, Bin Zhang

**Affiliations:** 1 Department of Gastroenterology, The Second Hospital of Nanjing, Nanjing University of Chinese Medicine, 1 Zhongfu Road, Nanjing, Jiangsu China; 2 Department of General Surgery, Jinling Hospital, Medical School of Nanjing University, Nanjing, China; 3 Department of Radiology, The Second Hospital of Nanjing, Nanjing University of Chinese Medicine, Nanjing, Jiangsu China

**Keywords:** Transnasal ileus tube, Obstructions, Crohn’s disease

## Abstract

**Background:**

Previous studies reported that transnasal ileus tube was a new and useful method for rapid relief of small intestinal obstruction. However, no study reported the impacts of the transnasal ileus tube for Crohn’s disease combined with intestinal obstruction. We aimed to describe the strategy to the small intestine obstruction caused by Crohn’s disease on the basis of transnasal ileus tube insertion.

**Methods:**

From November 2019 to November 2021, the data of 6 hospitalized patients with CD, diagnosed and conservatively treated in The Second Hospital of Nanjing, were not relived and retrospectively collected. After the insertion of transnasal ileus tube, demographic information, clinical features and treatment data were extracted from medical records.

**Results:**

Six Crohn’s disease patients with intestinal obstruction were included. Half of them were male. The patients aged from 29 to 70 years. Five patients had chronic intestinal obstruction more than one year. Three patients had intestinal surgery history. One patient had colonic abdominal fistula and anastomotic fistula, when she took intermittent usage of sulfsalazine and steroid. On admission, all the patients had abdominal pain, distention and mass. Five patients had anemia, low albumin and cholinesterase. All CDAI scores were more than 400. Compared to 19 patients with incomplete intestinal obstruction improved by nasogastric decompression tube, 6 patients with intestinal obstruction catheter had significant difference in time for relieving abdominal pain and distension (p = 0.003), time for alleviating abnormal mass (p ≤ 0.01), drainage volume (p = 0.004), and preoperative CDAI score (p = 0.001). Compared with X-ray image before insertion, complete remission of obstruction of 5 patients were observed in intestinal cavity after insertion. After 1–2 months nutrition, all the patients had small intestine resection and ileostomy, half of them underwent colectomy and fistula repair, and 4 patients were performed enterolysis at the same time, the residual small intestine length ranging from 250 to 400 cm. 1 patient had permanent ileostomy;1 patient had abdominal infection after operation. The typical manifestations of acute and chronic inflammation, transmural inflammation, pseudopolyps and serous fiber hyperplasia could be seen in pathological findings of patients 1 to 5. All the patients continued enteral nutrition after surgery. Four patients were treated with infliximab or vedolizumab.

**Conclusion:**

The current intestinal obstruction catheter which is used to treat patients with Crohn's combined obstruction can afford quick clinical remission, longer nutrition time, and suitable preoperative CDAI score for operation, which is worthy of wildly being used.

## Background

Crohn's disease, one of chronic autoimmune disease, is typically characterized by intestinal transmural damage. The key features for diagnosing CD comprises a combination of radiographic, endoscopic and pathological findings demonstrating focal, asymmetric, transmural or granulomatous features. Intestinal lesions include hyperplasia, stenosis, ulcerative bleeding, transmural perforation, etc. Crohn's disease, is usually complicated with obstruction, especially chronic incomplete obstruction, which required surgical operation. The perioperative principle of Crohn's disease is different from the enhanced recovery after surgery or fast track surgery of general gastrointestinal tumors. If there are no life-threatening complications, the operation should not be performed too hasty. Because of persistence of intestinal lesions and the incurability of surgery, the preoperative optimization strategy is expected to reduce postoperative complications, preserve intestinal segments, solve the complications of Crohn's disease, avoid the risk of short bowel syndrome, achieve the purpose of remission, improve nutritional status, reduce drug dependence, and improve the quality of life [1].

Surgical operation should be avoided at the active stage of Crohn's disease. During the perioperative period, they should withdraw steroid dependence as much as possible and ablate the use of hormones, immunosuppressor and other drugs. If the disease condition is stable, and the Crohn's disease activity index score(CDAI) is less than 150, qualitative surgical treatment can be carried out [2].

The perioperative strategy of incomplete obstruction need stop using the medication that can control Crohn's activity, relieve the obstruction as soon as possible, improve the nutritional status of patients, and provide surgical conditions for the optimization of surgery. Conservative management is the preferred option in the absence of peritonitis, including bowel rest, intravenous fluid therapy,and gastric decompression [3]. The nasogastric decompression tube can only be placed in the stomach and has no direct decompression effect on the contents of the small intestine, so it can not achieve the ideal remission effect [4]. Transnasal intestinal obstruction tube is a new method for rapid relief of intestinal obstruction in Crohn's disease complicated with small intestinal obstruction. In contrast to nasogastric decompression tube, the complete decompression site of the transnasal intestinal obstruction tube is directly above the obstruction plane of the proximal small intestine. Inhalation and decompression of gas and intestinal contents at the obstruction site can quickly alleviate the symptoms of small intestinal obstruction [5]. Resection and anastomosis of obstructive lesions can be successfully performed on most patients after preoperative preparation through catheter [6, 7]. This study describes the treatment, that is, surgical procedure on the basis of transnasal ileus tube insertion, of 6 cases of Crohn's complicated with small intestinal obstruction.

## Methods

### Population and exclusion criteria

**Fig. 1:**
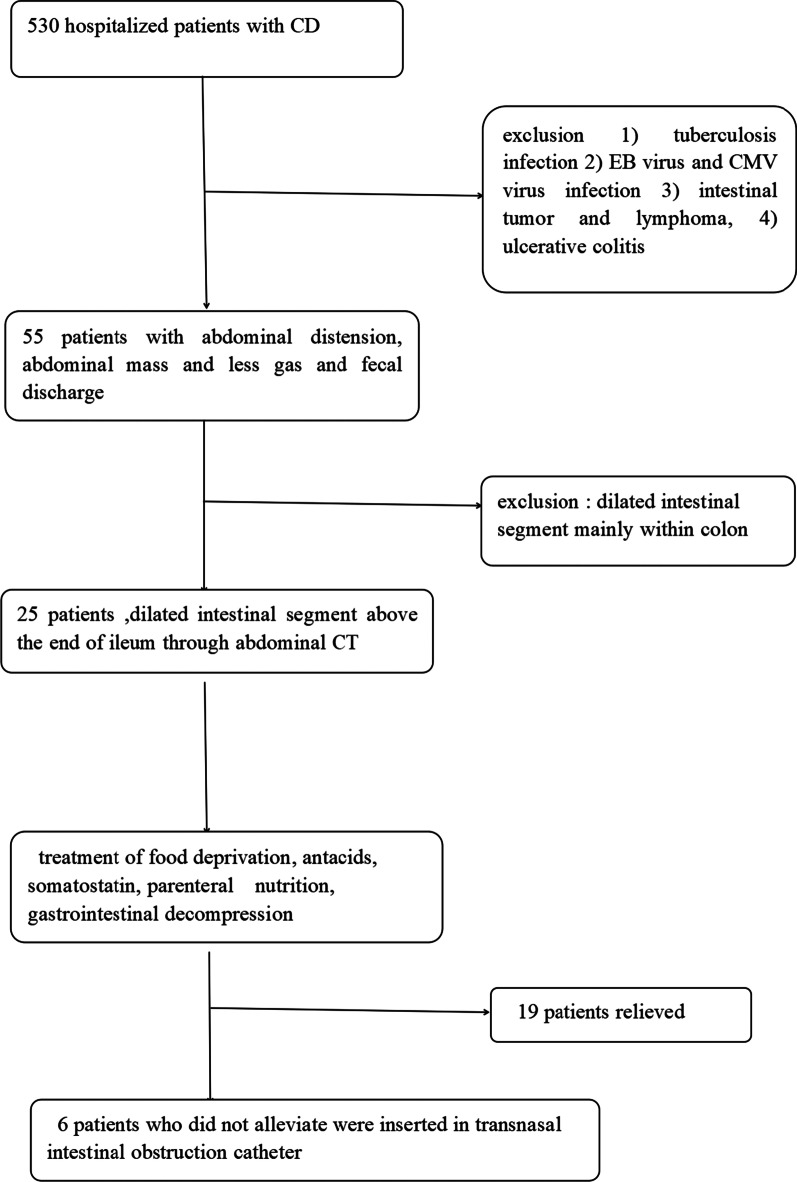
Process of cases screening

From November 2019 to November 2021, the data of 530 hospitalized patients with CD, diagnosed by clinical manifestations, radiographic and endoscopic findings in The Second Hospital of Nanjing, were retrospectively collected. At the same time, the following conditions were excluded: (1) tuberculosis infection, (2) EB virus and CMV virus infection, (3) intestinal tumor and lymphoma, (4) ulcerative colitis. Among 55 patients with abdominal distension, abdominal mass, less gas discharge and defecant discharge, 25 patients were confirmed to have intestinal obstruction according to dilated intestinal segment above the end of ileum through abdominal CT screening. 19 patients relieved after they were treated through the nasogastric decompression tube, food deprivation, antacids, somatostatin, and parenteral nutrition. 6 patients inserted transnasal intestinal obstruction catheter without remission. The screening process can be seen in Fig. 1.

### Procedure

The clinical data of 6 patients were firstly reviewed, then the transnasal intestinal obstruction catheter (CLINY Ileus Tube suite, Create Medic, Tokyo, Japan. The tube is 300 cm in length, 16 Fr or 18 Fr) [8] were implanted into small intestine as deep as possible under endoscopic guidance. After 24–48 h, the catheter depth can be adjusted according to radiography result so that the catheter can be located near the obstruction to help decompress. The next step is to collect the data which includes relief time of clinical symptoms, daily drainage volume, nutritional mode, NRS2002 score, and CDAI score before insertion. 1–2 months after transnasal ileus tube insertion, all the patients underwent individual preoperative evaluation and corresponding resection operation which were performed by senior expert groups of department of General Surgery at Jinling Hospital of Medical School of Nanjing University between January 2020 and October 2021. Surgical specimen were pictured and observed through microscopy after hematoxylin–eosin staining. Meanwhile, postoperative treatment were recorded.

### Laboratory and radiologic information

The laboratory tests included blood routine tests, liver function, PT, inflammatory biomarkers. Abdominal enhanced computed tomography (CT), X-ray or digestive tract radiography were performed for each patient and the results were collected.

### Statistical analysis

The continuous variables were presented as medians (interquartile range (IQR)). Categorical variables were shown as the counts. The independent group t tests or Mann–Whitney *U* were used to compare continuous variables between groups. Chi-square or Fisher exact test was used for the comparisons of categorical variables. P < 0.05 was considered as statistically significant. All data analysis was performed using SPSS version 22.0 software (SPSS Inc., Chicago, IL, United States).

## Results

### Clinical characteristics of the patients

The medical history of the patients were presented in Table 1. Half of the patients were male. The patients aged from 29 to 70 years. One patient had breast tumor and received the corresponding treatment. All the patients had no cigarette and alcoholic history. The clinical characteristics before and after occurrence /aggravation of intestinal obstruction were described (Tables 2,3). 5 patients had a history of taking immunosuppressive agents and steroids for more than 1 year, but stopped the medication on their own willing. We mark the immunosuppressant intake for more than 1 year as YES, and no less than 1 year as NO, and the discontinuous drugs for more than half a year and retaking for more than half a year as intermittent.

**Table 1:** The demographic characteristics and medical history of 6 patients

Characteristics	Patient1	Patient 2	Patient 3	Patient 4	Patient 5	Patient 6
Age	39	29	55	57	36	70
Sex	M	M	F	F	M	F
Anal fistula	No	No	No	No	Yes	No
Anal stenosis	No	No	Yes	No	Yes	No
Gallstones	No	No	No	No	Yes	No
Kidney stones	No	No	No	No	Yes	No
Ureterectasis	No	No	No	No	Yes	No
Abdominal abscess	No	No	Yes	No	Yes	No
Fatty liver	No	No	Yes	No	No	No
Malnutrition	No	No	No	Yes	No	No
Anemia	No	Yes	Yes	Yes	No	No
History of tumor	No	No	No	Breast cancer	No	No
History of non-intestinal surgery	No	No	No	Mastectomy	No	No

**Table 2:** Clinical characteristics before occurrence/aggravation of intestinal obstruction

Characteristics	Patient1	Patient 2	Patient 3	Patient 4	Patient 5	Patient 6
Diet	Normal diet	Element diet	Enteral nutrition transition to normal diet	Normal diet	EN transition to ordinary diet	normal diet
Intermittent EN	No	Yes	Yes	No	No	No
Abdominal pain, abdominal distension	Intermittent	Intermittent	Intermittent	Recurrent	Intermittent	Intermittent
Vomit	No	No	No	Recurrent	No	No
Bloody stool	No	No	No	No	Yes	No
Abdominal mass	No	No	Yes	No	Yes	No
Abdominal scars	Yes	No	Yes	No	Yes	No
Ascites	No	No	No	No	Yes	No
CD diagnosed years	2 W	4Y	15Y	12Y	16Y	23Y
Chronic intestinal obstruction time	3 W	3Y	2Y	11Y	1Y	4 M
History of intestinal surgery (years)	6Y	No	18Y first time 14Y second time	NO	7Y	No
Excision site	appendix	No	Small intestine 1 m, ileum 30 cm, transverse colon 20 cm	No	Right hemicolon, small intestine	No
Intestinal perforation	No	No	No	No	Yes	No
Intestinal fistula						
Internal fistula	No	No	Colonic abdominal fistula, anastomotic fistula	No	No	No
External fistula	No	No	No	No	No	No
Treatment						
Mesalazine	No	Yes	No	Yes	Yes	No
Sulfasalazine	No	No	Intermittent	No	Yes	No
Steroid	No	No	Intermittent	Yes	Yes	No
Biological agents	No	No	No	Trastuzumab	No	No
Azathioprine	Yes	No	Yes	Yes	Yes	No

**Table 3:** Clinical characteristics on the admission of 6 patients

Characteristics	Patient1	Patient 2	Patient 3	Patient 4	Patient 5	Patient 6
Occurrence/aggravation of intestinal obstruction	3 W	1 M	1 M	1Y	2 W	1 M
Abdominal pain and distension	Yes	Yes	Yes	Yes	Yes	Yes
vomit	No	No	Yes	No	No	No
Bloody stool	Yes	No	No	No	No	No
Abdominal mass	No	Yes	Yes	Yes	Yes	No
Abdominal scar	Yes	No	Yes	No	Yes	No
Ascites	Yes	Yes	No	Yes	Yes	No
Fever	No	No	No	No	No	No
Intestinal perforation	No	No	No	No	Yes	No
Intestinal fistula						
Internal fistula	Small intestinal colonic fistula	No	No	Small intestine bladder fistula	No	No
External fistula	No	No	No	No	No	No
Treatment						
Switch EN to fasting	No	Yes	No	No	No	Yes
PN	Yes	Yes	Yes	Yes	Yes	Yes
Mesalazine (used before fasting)	Yes	No	No	No	No	No
Somatostatin	Yes	Yes	Yes	Yes	Yes	Yes
Antibiotics	No	No	No	Yes	Yes	No
Switch Prednisone to NONE	No	No	No	No	Yes	No

2 patients had intermittent enteral nutrition (EN) before admission. All the patients stopped EN or regular diet or oral medication when intestinal obstruction was diagnosed. Then all the patients took total parenteral nutrition (TPN), and after insertion they took Partial Enteral Nutrition (PEN) on the basis of their tolerance as soon as possible.

### Laboratory and image abnormalities

**Fig. 2:**
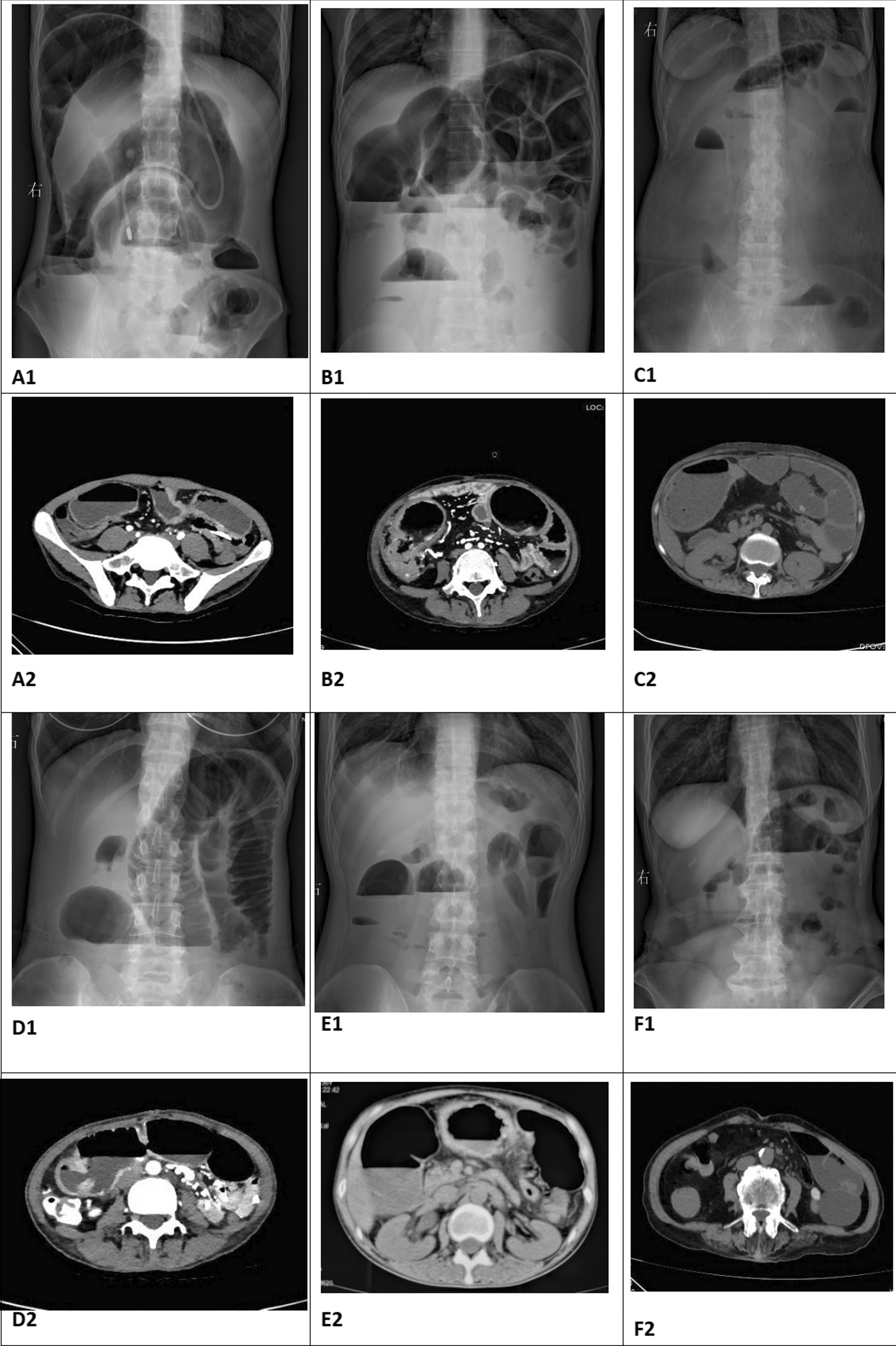
Abdominal X-Ray and CT at the beginning of obstruction confirmation. **A1** and **A2** showed the obstruction sign before tube insertion of patient 1; **B1** and **B2** showed the showed the obstruction sign before tube insertion of patient 2; **C1** and **C2** showed the obstruction sign before tube insertion of patient 3; **D1** and **D2** showed the obstruction sign before tube insertion of patient 4; **E1** and **E2** showed the obstruction sign before tube insertion of patient 5; **F1** and **F2** showed the obstruction sign before tube insertion of patient 6

After admission, 6 patients took blood examination, abdominal X-ray and CT. NRS2002 score and CDAI score were calculated. Five patients(83.3%) had anemia low ALB level, and all the patients had increasing fecal calprotectin and high CDAI scores(seen in Table 4. All the patients had typical Characteristic imaging changes of intestinal obstruction (seen in Fig. 2).

**Table 4:** Laboratory abnormalities at the beginning of this hospitalization

Characteristics	Patient1	Patient 2	Patient 3	Patient 4	Patient 5	Patient 6
WBC(3.69–9.16 × 10^9^/L)	2.26	5.66	3.49	3.93	7.47	5.33
NE (2–7 × 10^9^/L)	1.66	3.4	2.42	2.59	5.66	3.01
LY (0.8–4 × 10^9^/L)	0.41	1.75	0.84	0.94	1.16	1.65
HGB (113–151 g/L)	97	89	88	83	105	136
PLT (101–320 × 10^9^/L)	238	362	237	234	290	206
CRP (0–10 mg/L)	13.11	12.36	10.3	54.16	47.7	3
PCT (0.01–0.052 ng/ml)	0.1	0.05	0.34	0.09	0.05	≤ 0.01
PT (14–21 Sec)	12.3	12.7	14.5	24.5	12.5	17.3
ESR (0–20 mm/h)	8	5	8	12	18	9
Fecal calprotectin (0–200 µg/g)	> 1800	> 1800	> 1800	> 1800	> 1800	> 1800
ALB (35–55 g/L)	31.8	18	33.7	33.6	27	38.9
TBIL (3–19 µmol)	4.3	9.8	8.8	8.7	7.3	4.4
DBIL (0.5–6.8 µmol)	2.4	5.2	4.6	5.5	4.2	1.2
CHE (4230–13,000 U/L)	3969	2066	2524	2305	1985	5770
Ca (2.1–2.6 mmol/L)	1.8	1.81	2.00	2.17	2.05	2.35
Fe (5.83–34.5 µmol)	4.47	18.23	12.99	4.08	9.6	18.72
NRS2002	12	10	12	10	12	10
CDAI	498.74	518.98	511.04	485.04	515.79	401.97

### Therapeutic efficacies

19 patients with incomplete intestinal obstruction improved by nasogastric decompression tube and 6 patients treated with intestinal obstruction catheter were selected to observe the relief of clinical symptoms, nutrition time, volume of drainage, and the variation of CRP, ESR, CDAI before surgery, as well as complication after operation. See Table 5. From that table, we can see significant difference in time for relieving abdominal pain and distension (*p* = *0.003*), time for alleviating abnormal mass (*p* ≤ *0.01*), volume of drainage (*p* = *0.004*), preoperative nutrition time (*p* ≤ *0.01)*, and preoperative CDAI scores (*p* = *0.001*).

**Table 5:** Comparison of two groups on therapeutic effect

Parameter	Group 1 (transnasal ileus tube n = 6)	Group 2 (nasogastruic tube n = 19)	Statistical value	*P*
Time for relieving abdominal pain and distension(day)	1.33 ± 0.516	2.68 ± 0.946	3.313	0.003
time for alleviating abnormal mass(day)	2.33 ± 0.516	4.74 ± 2.156	4.470	≤ 0.01
Volume of drainage(ml/day)	666.67 ± 250.333	167.37 ± 76.583	4.815	0.004
Preoperative nutrition time(week)	6.67 ± 1.506	2.05 ± 0.970	8.886	≤ 0.01
CRP	4.17 ± 1.472	5.95 ± 4.116	1.026	0.315
ESR	8.17 ± 2.858	14.42 ± 10.249	1.457	0.159
Preoperative CDAI score	163.48 ± 30.14	231.20 ± 26.43	4.937	0.001
Postoperative complications	1 (16.7%)	6 (31.6%)	0.483	0.487

**Fig. 3:**
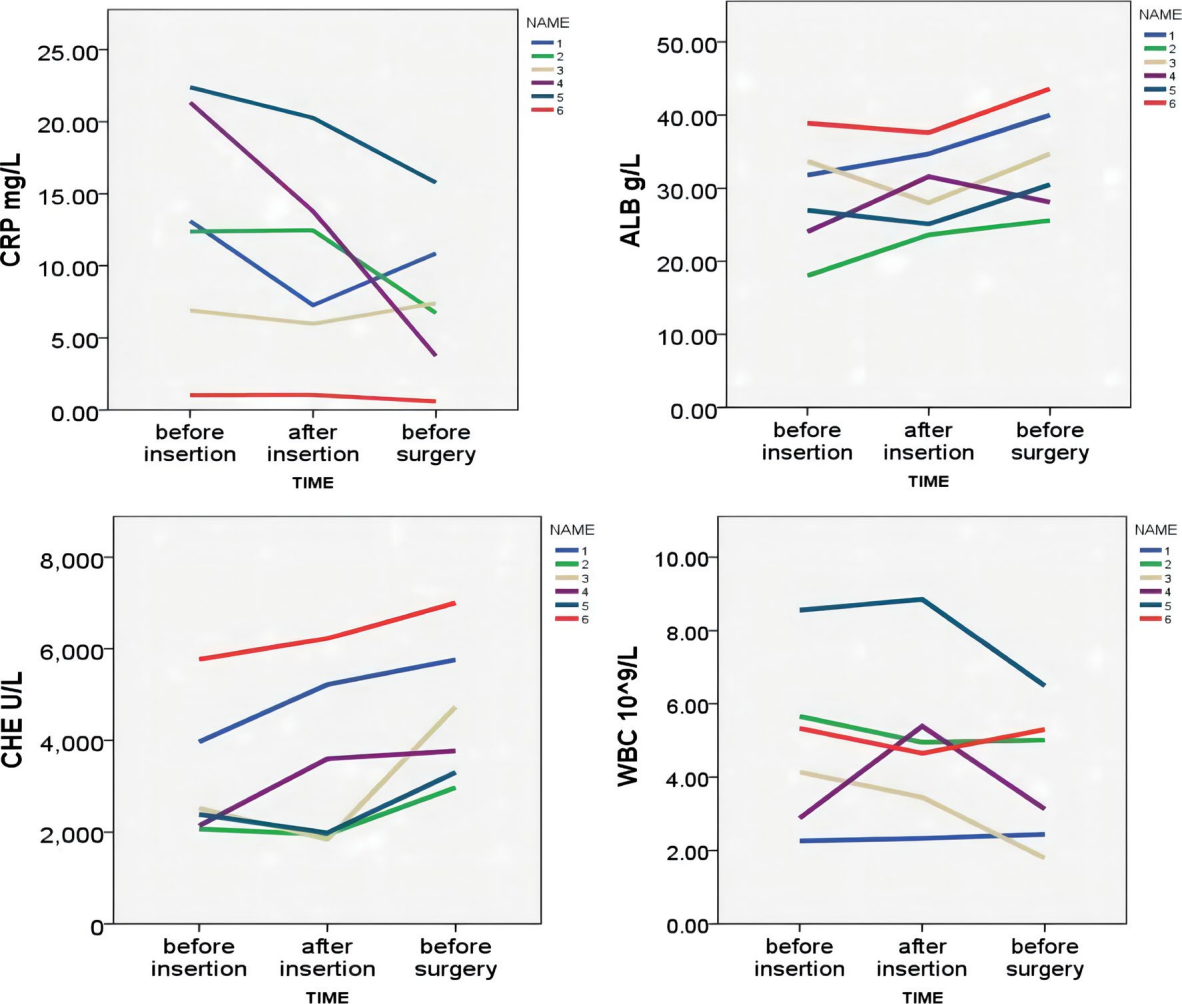
Parameters comparison of 6 patients before catheter insertion, 1W after insertion and before surgery

For 6 patients, the blood examinations were performed before tube insertion, before and after surgical operation so as to investigate the variation of CRP, ALB, CHE, WBC (seen Fig. 3).

The abdominal X-ray was taken 2-4 weeks after the insertion of intestinal obstruction catheter. The digestive radiography examinations were performed before operation.

**Fig. 4:**
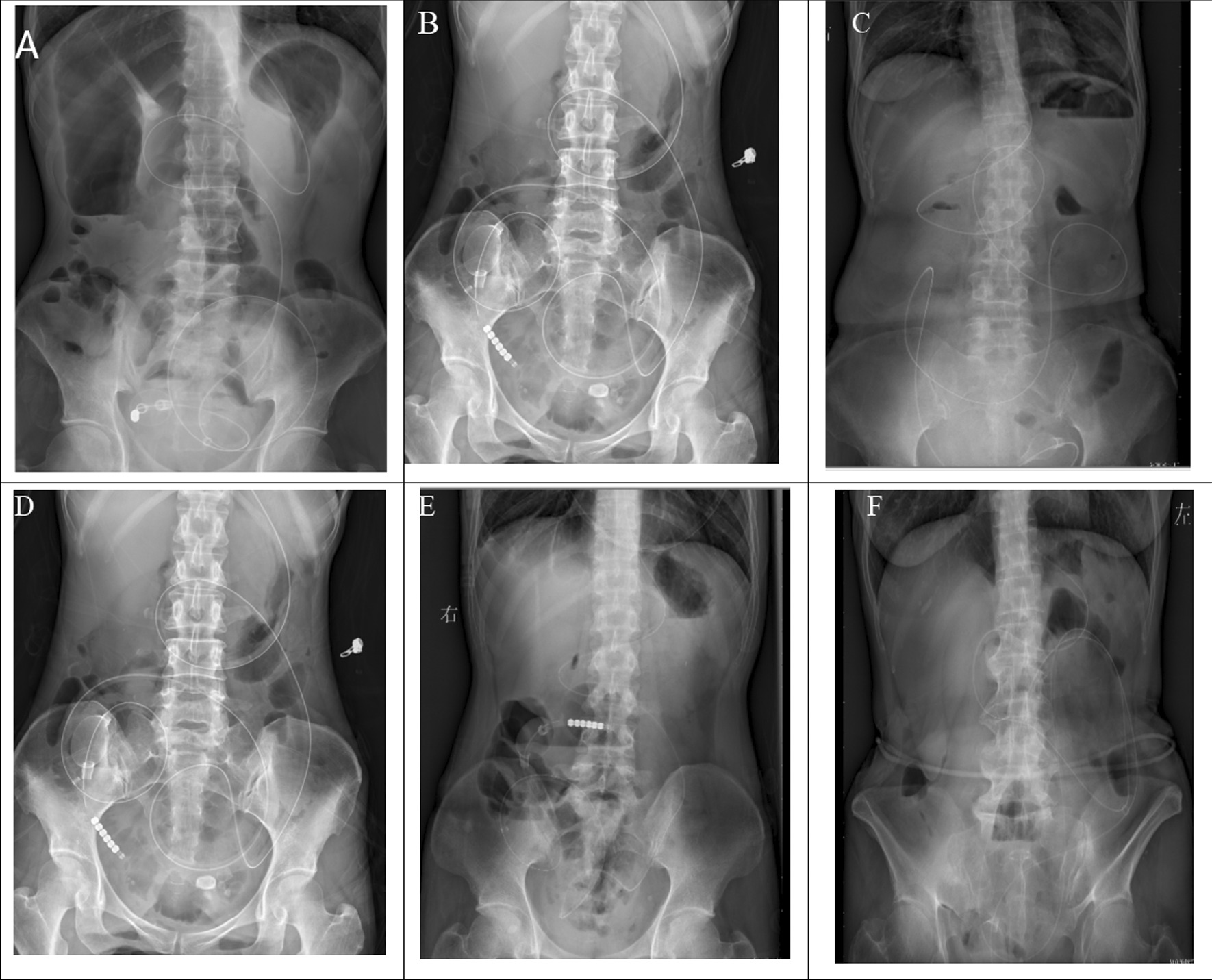
Relief after 2w-4w transnasal ileum tube insertion by X-ray.** A** image of X-RAY after transnasal ileum tube insertion of patient 1; **B** image of X-RAY after X-RAY of patient 2; **C** image of X-AY after transnasal ileum tube insertion of patient 3; **D** image of X-AY after transnasal ileum tube insertion of patient 4; **E** image of X-AY after transnasal ileum tube insertion of patient 5; **F** image of X-AY after transnasal ileum tube insertion of patient 6. **B**–**F** indicated fully relieved; **A** indicated partially relieved

**Fig. 5:**
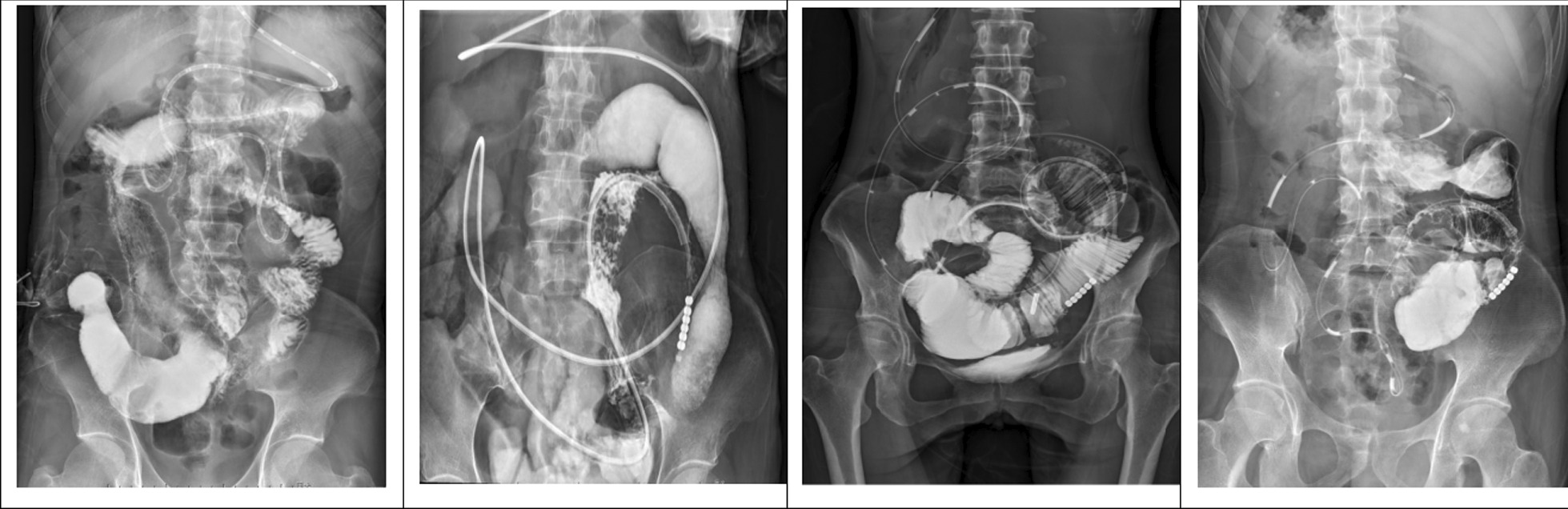
Gastrointestinal radiological findings of stricture location of before surgery. From left to right: Patient 1, Patient 3, Patient 4, Patient 5. P1: segmental ileal stenosis, P3: multiple dilatation and segmental stenosis of small bowel, P4: ileal stenosis above ileocecal region, P5: stricture of ileocolic anastomosis, dilated ileum

Except for patient 1, the other 5 patients were found obvious alleviation (seen Fig. 4). Part of the patients (4/6) had gastrointestinal radiography to make the obstruction position clear (Fig. 5). The intestinal obstruction of P1 alleviated partially due to multiple segmental stenosis which could be observed by gastrointestinal radiography.

### Therapeutic outcome of surgery and Postoperative treatment

**Fig. 6:**
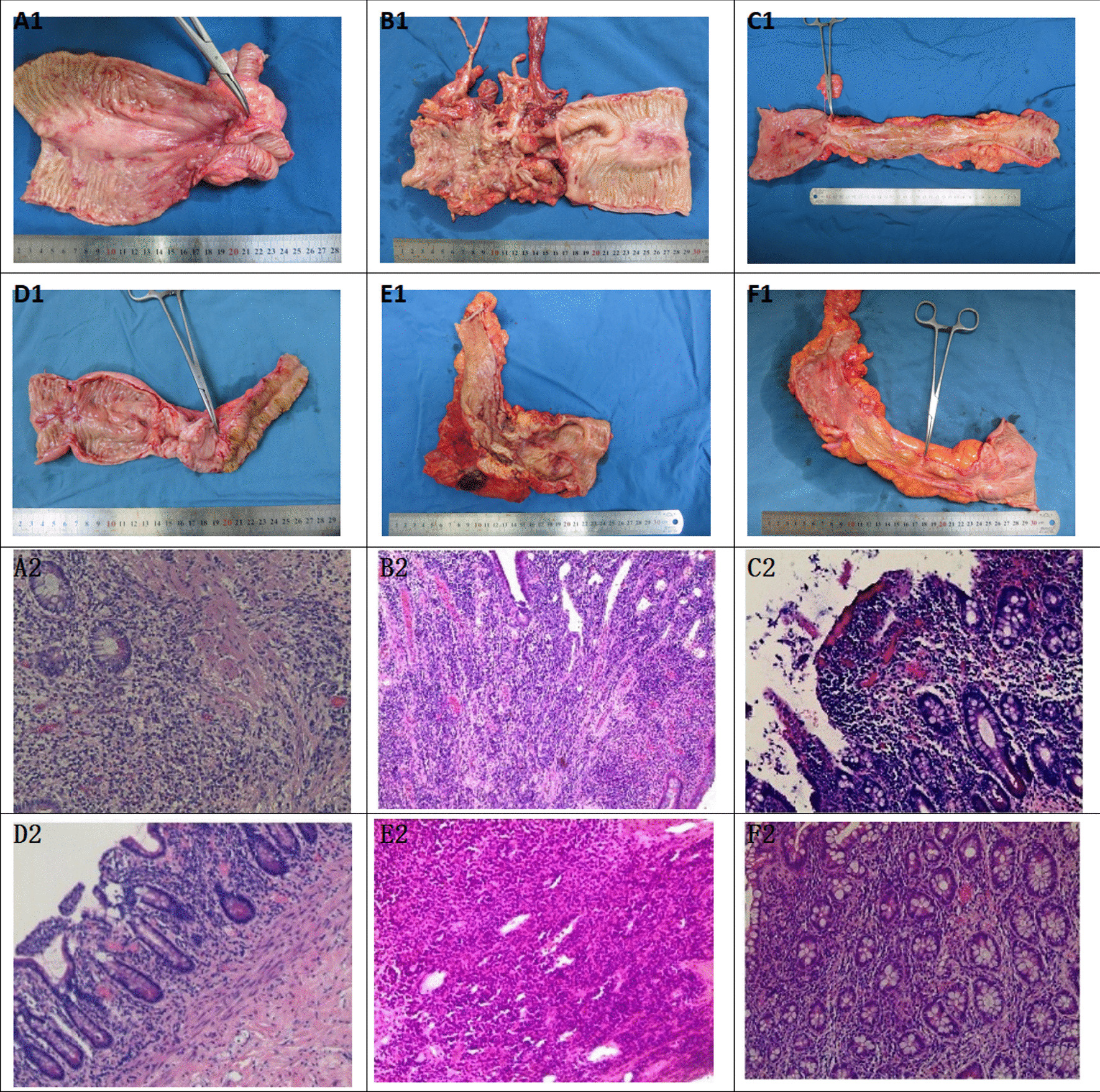
Macroscopic and microscopic findings of excisional intestinal specimen of 6 patients. **A1** and **A2** macroscopic and microscopic findings of patient 1; **B1** and **B2** macroscopic and microscopic findings of patient 2; **C1** and **C2** macroscopic and microscopic findings of patient 3; **D1** and **D2** macroscopic and microscopic findings of patient 4; **E1** and **E2** macroscopic and microscopic findings of patient 5; **F1** and **F2** macroscopic and microscopic findings of patient 6

6 patients were performed different optimal operation in accordance with their different condition. Then macroscopic and microscopic findings of their own intestinal resection were observed, as shown in Table 6 and Fig. 6.

**Table 6:** Nutrition time, operative methods, postoperative pathology and postoperative treatment of 6 patients

Characteristics	Patient1	Patient 2	Patient 3	Patient 4	Patient 5	Patient 6
Retention time of intestinal obstruction catheter (week)	4	7	4	6	3	6
Reasons for pulling out of intestinal obstruction catheter	Throat pain	Surgery	Throat pain	Throat pain	Throat pain	Surgery
Nutrition period after tube insertion(week)	8	7	6	7	4	6
Operative methods						
Small intestine resection	Yes	Yes	Yes	Yes	Yes	Yes
Colectomy	No	No	Yes	No	Yes	Yes
Residual small intestine length	300 cm	400 cm	300 cm	250 cm	290 cm	300 cm
Intestinal lysis	Yes	No	Yes	Yes	Yes	No
Fistula repair	Yes	No	No	Yes	Yes	No
Ileostomy						
Stoma number	2	1	1	2	1	1
Permanent	No	No	Yes	No	No	No
Closure enterostomy	Yes	No	No	Yes	No	No
Postoperative fistula	No	No	No	No	No	No
Incision infection/abdominal infection	No	Yes	No	No	No	No
Postoperative pathology						
Acute an chronic inflammation	Moderate-severe	Moderate-severe	Moderate	Moderate	Moderate	Moderate
Transmural inflammation	Yes	Yes	Yes	No	Yes	No
Pseudopolyp	Yes	Yes	No	Yes	Yes	Yes
Ulcer	Superficial	Transmural	Superficial	No	Transmural	No
Serous fibrous hyperplasia	No	Yes	Yes	Yes	Yes	No
Postoperative treatment						
Immunosuppressant	No	No	AZA for 0.5y Thalidomide for 1y	No	No	No
EN	Yes	Yes	Yes	Yes	Yes	Yes
Biological agents	Infliximab (start form 1 mon after surgery)	No	Vedolizumab (start form 1 year after surgery)	Vedolizumab (start form 4 mon after surgery)	NO YET	Vedolizumab (start form 1 mon after surgery)
Follow-up postoperation	13M	Lost	3M	7M	2W	2M

All the patients had small intestine resection and ileostomy, half of whom underwent colectomy and fistula repair. Meanwhile, 4 patients were performed intestinal lysis, with residual small intestine length ranging from 250 to 400 cm. 1 patient had permanent ileostomy;1 patient had abdominal infection after operation.

The typical manifestations of acute and chronic inflammation, transmural inflammation, pseudopolyps and serous fiber hyperplasia can be seen in pathological findings of patients 1 to 5. All the patients continued enteral nutrition after surgery. Four patients were treated with infliximab or vedolizumab.

## Discussion

The benefits of surgical treatment of Crohn's disease have been greatly improved. However, it remains challenging for surgeons because gastrointestinal surgery is still a high-risk operation for Crohn's disease patients [9]. Most patients with Crohn's disease have obvious severe symptoms when the urgent operation is needed, many of whom were even misdiagnosed or delayed being diagnosed for a long time. The physiological homeostasis environment function in vivo disordered, and the surgical complications such as obstruction, bleeding and fistula further make the body function worsen. In addition, patients with Crohn's disease often suffered immune dysfunction, infection, anemia, malnutrition, and they usually took many immunosuppressants and biological agents. All the complication and therapeutic destroyed the immune function and increased the risk of postoperative complications [10]^.^ Therefore, the perioperative optimization strategy to reduce postoperative complications, avoid future recurrence and maintain postoperative remission is particularly important. A logistic regression analysis of one study was revealed that operative age and preoperative EN were independent risk factors for postoperative complications [10]. A number of studies [11–14] have also confirmed that preoperative parenteral nutrition support can significantly reduce postoperative complications, and the nutritional status and inflammation of patients can be improved. The research results of Grivceva et al. [15] suggested that the use of TPN had no obvious relationship with postoperative complications, but indicated the positive significance of PN for surgical optimization in different ways. Therefore, prolonging the nutritional time as long as possible is an important treatment strategy in the perioperative period.

Six patients with Crohn's disease were followed up. Five of them were definitely diagnosed with CD several years ago. One of them just diagnosed Crohn's disease with intestinal obstruction after this admission. Three patients with incomplete obstruction had a history of abdominal surgery several years ago, and P3 had multiple operations due to recurrent intestinal obstruction. Five patients took the different immunosuppressive medications to control disease activity. In spite of the usage of strong immunesuppressive drugs, CD patients frequently experience the recurrence and over half will have an intestinal resection within 10 years after diagnosis, one-third undergo a repeat resection within 5 years [16]. Different from general gastrointestinal diseases, The resection of the intestines of patients with Crohn's disease at the lesion site can not completely cure the disease and will eventually inevitably relapse [17], while the intestinal fistula and other diseases during abdominal surgery are active factors for incomplete obstruction [18]. Preoperative treatment measures are to maximize the effect of postoperative resection remission, prolong the recurrence cycle, reduce the recurrence rate and reduce perioperative complications. With the proposal of nutritional immunity and nutritional ecology, nutritional support plays an important role in intentional rehabilitation. These 6 patients were mainly took element diet and normal diet before the onset of intestinal obstruction. The nutritional status of the patients were usually poor after obstruction, and there were weight loss, anemia, albumin, cholinesterase reduction, low iron and calcium. In order to reduce intestinal secretion and protect intestinal mucosa, all the patients fasted, took parenteral nutrition, and were injected somatostatin. After the placement of intestinal obstruction catheter, except for P1, intestinal dilatation of other five patients obviously relived to different extents, which could be seen from X-ray image. Through angiography, P1 was observed multiple segmental stenosis before surgery which possibly resulted into partially-relieved obstruction. Two patients used enteral combined parenteral nutrition to improve nutritional status. Studies have shown that the usage of total enteral nutrition for 3 months before operation can significantly increase the nutritional reserve, reduce adverse reactions and reduce the incidence of postoperative complications in patients of Crohn's with intestinal fistula [11]. For CD patients presenting with acute small-bowel obstruction without bowel ischaemia or peritonitis, deferred surgery is the preferred option [3], so all six patients took comparatively long time to improve the nutrition condition before surgery in order to decrease the incidence of adverse surgical outcome. Regarding intestinal stricture treatment, endoscopic balloon dilation(EBD) and surgical treatment can be both considered as one of the options. Studies have shown EBD is more effective and safer for a small number (< 4) or short segment (< 4-5 cm) of strictures in a close proximity, while multiple strictures or long segment strictures(> 5 cm) of bowel may benefit more from surgical resection, anastomosis or stricturoplasty as a result of the involvement of stenotic bowel angulation [19]. That’s the reason why the six patients underwent surgery to relieve the stenosis.

It is knonwn that malnutrition is an independent dangerous factor for adverse postoperative outcomes of CD. Therefore, oral or enteral nutrition is the priority of nutritional support;[20]. Compared with 19 patients with nasogastric tube decompression whose nutrition time is about 2 weeks, the nutrition time of the 6 patients with transnasal ileus tube is about 1–2 months which was beneficial to intestinal resection. however, the period of nutrition was still less than 3 months, which may be due to 1.after the insertion, enteral nutrition is usually fed through the intestinal obstruction catheter. The position of enteral nutrition is close to the obstruction site, and there are relatively less enteral tubes that can effectively absorb enteral nutrition. 2. The intestinal obstruction catheter is too thick for some patients to tolerate the long-term compression of the nasal cavity and throat, resulting in a short retention time of the obstruction catheter and affecting the time of enteral nutrition. 3. The site of obstruction is mostly located in the small intestine, and the effective area of enteral nutrition absorption is restricted, which affects enteral nutrition. 4. In addition to obstruction, some patients also have internal fistula. For patient 1 and patient 4 and 5, nutrition infusion to the small intestine may enter into colon or bladder fistula, affecting the absorption of the small intestine, which is not conducive to the healing of fistula and has an adverse impact on the correction of patients' nutritional status. To solve those dilemma, the nasogastric tube or nasointestinal tube may be inserted to nutrition the proximal small intestine in addition to the small intestinal decompression tube. However, if the patient has gastric or duodenal fistula, the nutrition effect is expected not to be satisfied. It is an important method to prolong the period of enteral nutrition as far as possible in the treatment of Crohn's patients with small intestinal obstruction. It is worth mentioning that there is no significant difference in postoperative complications,the results of CRP and ESR between the two groups of patients with different decompression tubes, possibly as a result of the small sample size.

A study [21] have shown that the way to combine mesalazine with enteral nutrition is effective in the treatment of active Crohn's disease, which can induce remission, improve the nutritional status and improve the surgical tolerance of patients. Five patients had used mesalazine or azathioprine before the onset of this disease, but enteral nutrition was not sustainable. In case of obstruction and internal fistula, patients stopped immunosuppressants, including azathioprine, thalidomide and hormones, corrected the nutritional status as much as possible, and resected the intestinal canal as little as possible during the operation, so as to avoid postoperative complications. The macrosopic and microscopic change of the intestinal resection are consistent with the typical manifestations of Crohn’s disease complicated with intestinal obstruction. The typical manifestations of acute and chronic inflammation, transmural inflammation, pseudopolyps and serous fiber hyperplasia can be seen in patients 1 to 5, and pseudopolyps can be seen in patients 6. Even there was no typical transmural inflammation, the diagnosis of Crohn’s disease still can not be excluded accrouding to the lesion location and clinical symptoms. Four patients were treated with infliximab or vedolizumab. One patient was lost to follow-up after operation, and one was currently observed after operation. Vedolizumab was planned to be given one month after operation. All patients continued EN for now and will gradually adjust to their appropriate diet in the future.

From the comparison of short-tube usage(recommended by all the CD-related guidelines present) group and long-tube application group, we can conclude the patients can still benefit from long-tube insertion even though the patients of short-tube insertion experience the failure of effective treatment of intestinal obstruction. Studies show that long tube decompression have succeeded in more than 70% of the patients with benign adhesive small bowel obstruction [22, 23]; This procedure has been regarded as a simple, safe and minimally invasive approach. As a result, it is recommended as the initial treatment for benign adhesive bowel obstruction [24]. However, short tube insertion treatment is not appropriate for complicated intestinal obstruction.

This article described the medical history, diagnosis, perioperative treatment, operation process, postoperative pathology and biological treatment of 6 Crohn’s patients with incomplete obstruction and they were treated with transnasal ileus tube by abstracting their medical record. There were several limitations to this study. Firstly, This was a single-centered, retrospective study. Secondly, the sample size is small. The results need to be confirmed by a larger sample study. In addition, follow-up time was not long enough to observe clinical outcomes. When we confronted with complicated cases of obstruction CD which is still a great challenge for experts of both gastroentergolgy and general surgery department, the treatment strategy may be adopted as followed, first step is rapid decompression, second condition is to prolong the nutrition time as much as possible and improve general condition, and third part is when CDAI scores increased as high as to meet the satisfaction to operation, final method is usage of biological agents and TPN to control the CD activity.

## Conclusion

By comparing the two groups of patients with small sample size, the current intestinal obstruction catheter which was used to treat patients with Crohn's combined obstruction could afford quick clinical remission, longer nutrition time, and suitable preoperative CDAI score for operation, which is worthy of being wildly used. More similar data should be collected and analyzed to draw statistically significant conclusions.

## Data Availability

All data generated or analyzed during this study are included in this published article.

## Abbreviations

WBC: White blood cell
ALB: : Albumin
CHE: : Cholinesterase
ESR: : Erythrocyte sedimentation rate
CRP: : C-reactive protein
PT: Prothrombin
